# Case report: Transvenous coil embolization of a high-grade Galenic dural arteriovenous fistula

**DOI:** 10.3389/fneur.2023.1128563

**Published:** 2023-04-11

**Authors:** Christiana M. Cornea, Nathan Quig, Edward Yap, Sten Y. Solander

**Affiliations:** ^1^University of North Carolina School of Medicine, Chapel Hill, NC, United States; ^2^Department of Neurosurgery, University of North Carolina Hospitals, Chapel Hill, NC, United States; ^3^Department of Radiology, University of North Carolina Hospitals, Chapel Hill, NC, United States

**Keywords:** dural arteriovenous fistula, vein of Galen, transvenous embolization, occluded straight sinus, endovascular coiling

## Abstract

**Introduction:**

Galenic dural arteriovenous fistulas (dAVFs) are a rare form of dAVF and rarely described in the literature. Their distinct location requires different surgical approaches than dAVFs occurring at the nearby sites of the straight sinus and torcular Herophili, and their high risk of hemorrhage makes these dAVFs very challenging to approach surgically. In this report, we present a unique case of Galenic dAVF.

**Case description:**

The patient is a 54-year-old female who presented with a 2-year history of progressive headaches, cognitive decline, and papilledema. A cerebral angiogram demonstrated a complex dAVF to the vein of Galen (VoG). She underwent transarterial embolization with Onyx-18 which resulted in minimal reduction in arterial venous shunting. She subsequently underwent a successful transvenous coil embolization resulting in complete occlusion of dAVF. The patient’s postoperative course was complicated by interventricular hemorrhage; however, she had a remarkable clinical recovery with resolution of headaches and improvement in cognitive function. A follow-up angiogram completed 6  months post-embolization demonstrated very mild residual shunting.

**Conclusion:**

In the unique case presented here, we demonstrate the efficacy of transvenous embolization *via* an occluded straight sinus as an alternative therapeutic option to eliminate cortical venous reflux.

## Introduction

Cerebral dural arteriovenous fistulas (dAVFs) are formed by the abnormal drainage of meningeal arteries directly into the dural venous sinuses or cortical veins (meningeal, subarachnoid) (1). Most are thought to be idiopathic in nature; however, associations with dural sinus thrombosis, venous hypertension, previous craniotomy, trauma, and infection have been reported (1, 2). They commonly present in the region of the transverse-sigmoid sinus, though several other locations have been identified (3, 4). Cerebral dAVF symptom severity is largely determined by venous drainage patterns, severity of shunting, and location. Cortical venous and pial venous reflux are known risk factors for hemorrhage, the most serious sequela in dAVF (5).

Dural AVFs at the vein of Galen (VoG) are a rare form of falco-tentorial dAVF and tend to have an aggressive course with a high risk of hemorrhage (2, 3). The deep midline location and fistulous connection at the VoG categorize them as Type 1, on the proposed classification of tentorial dAVFs by Lawton et al. (6). Given the location, VoG dAVF’s presenting with hemorrhage can have devastating outcomes. Their distinct location at the anterior falco-tentorial junction and complex venous drainage (often earning a Borden Type III classification), make them among the most difficult fistulas to approach surgically (2, 5, 7). Transvenous approach around the tentorium to deeper locations of the brain, particularly for tentorial dAVFs that drain directly in the subarachnoid veins, is challenging (6). In this report, we present a case of dAVF with fistula point at the VoG that was successfully treated by transvenous embolization.

## Case description

**Figure 1:**
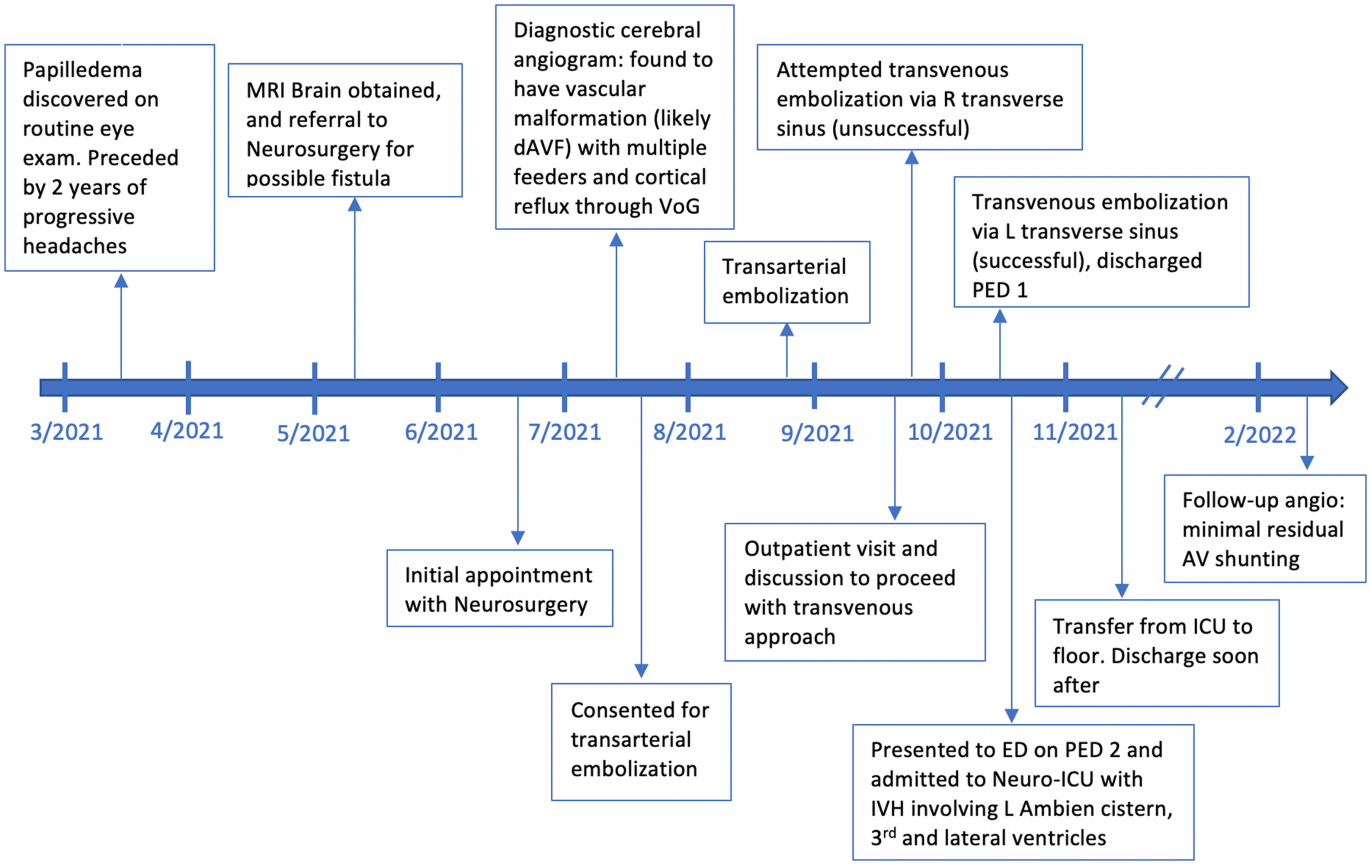
A timeline of events beginning with initial discovery of papilledema, outpatient visits related to the dAVF, angiography, embolization events, adverse events, and follow-up.

Our patient is a 54-year-old female with a history of chronic back pain, obstructive sleep apnea (OSA), depression, hypertension and hyperlipidemia, visual impairment requiring corrective lenses, and worsening headaches who was found to have papilledema on a routine ophthalmologic exam. She additionally has a family history of stroke in both maternal grandparents and aneurysms in both paternal grandparents. On presentation she reported 2 years of progressive headaches and subjective cognitive decline. Physical examination revealed no neurologic deficits except for mild give away weakness in bilateral lower extremities secondary to back pain. A brain MRI was obtained which demonstrated diffusely engorged cortical veins and a severely dilated left superior ophthalmic vein which was concerning for a dAVF or cavernous carotid fistula. A timeline of events from initial presentation through treatment and follow-up can be found in Figure 1.

**Figure 2:**
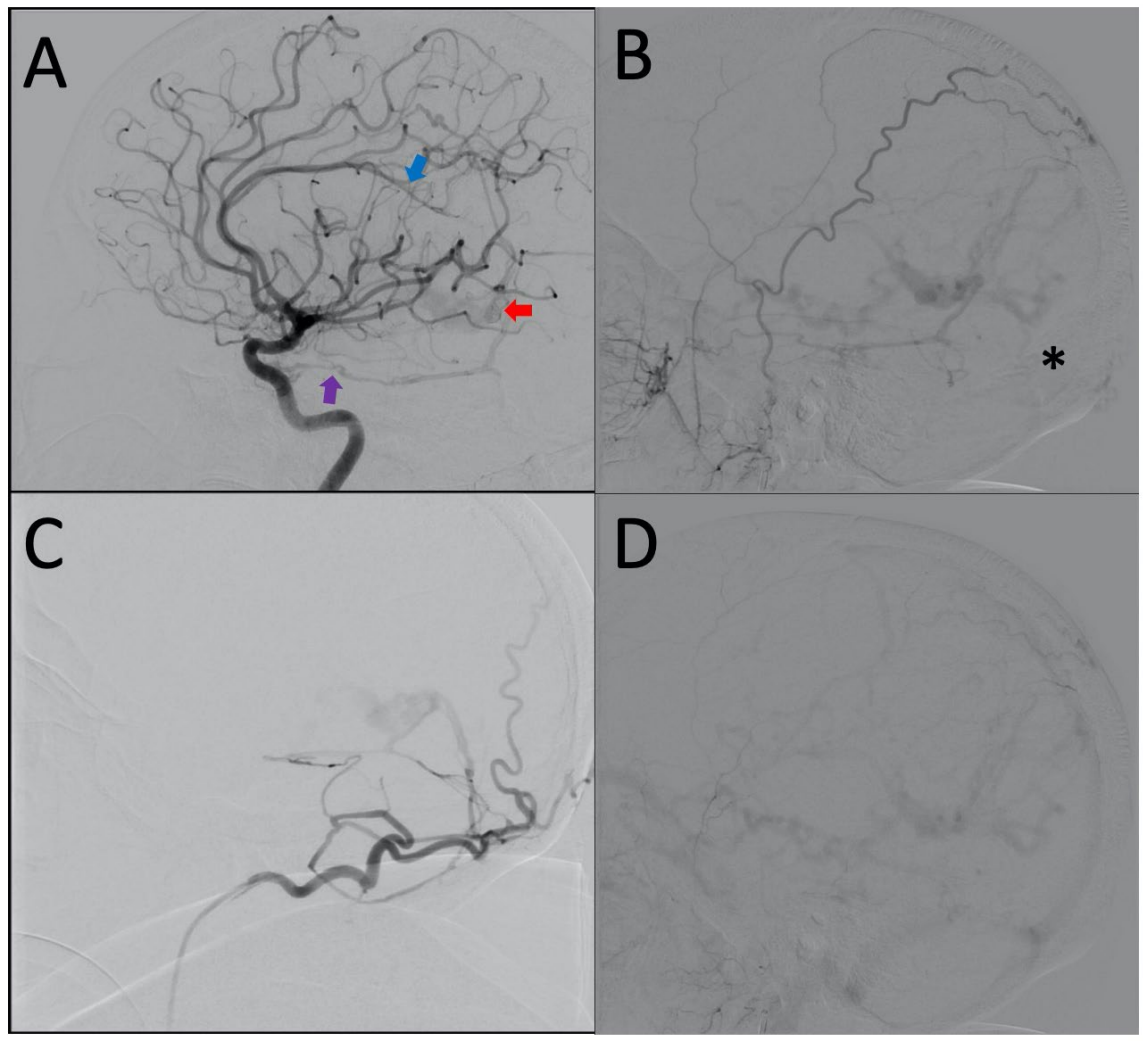
**(A)** Lateral projection of the right ICA demonstrating early shunting from the tentorial branch of the MHT (purple arrow) and distal ACA (blue arrow) filling the dilated vein of Galen (red arrow). **(B)** Lateral projection of the right ECA showing retrograde venous drainage and occlusion of the straight sinus (black asterisk). **(C)** Lateral projection of the right occipital artery. **(D)** Lateral projection of right ECA, late venous phase, demonstrating generalized venous congestion.

A cerebral angiogram (Figures 2A–D) was obtained which demonstrated a complex dAVF with the fistulous connections at the vein of Galen (Lawton et al. Type 1). The arterial supply of the dAVF came from several arteries including bilateral superficial temporal arteries, bilateral occipital arteries, bilateral tentorial branches of the meningohypophyseal trunk, and bilateral branches of the posterior cerebral artery. There was a complex deep venous drainage pattern with retrograde venous drainage from the vein of Galen anteriorly to the left superior ophthalmic vein through a severely dilated basal vein of Rosenthal, and corticovenous reflux through the internal cerebral veins to several dilated medullary veins. The straight sinus was occluded and there was partial occlusion of the left transverse sinus. It is possible that the occluded straight sinus was the inciting event leading to this complex dAVF.

The treatment options were discussed including: transarterial embolization, transvenous embolization, open surgical ligation, radiosurgery, and continued observation. After a thorough discussion with the patient and her family, we decided to proceed with transarterial embolization, knowing that this dAVF would likely require multiple embolizations or a different approach for complete treatment.

**Figure 3:**
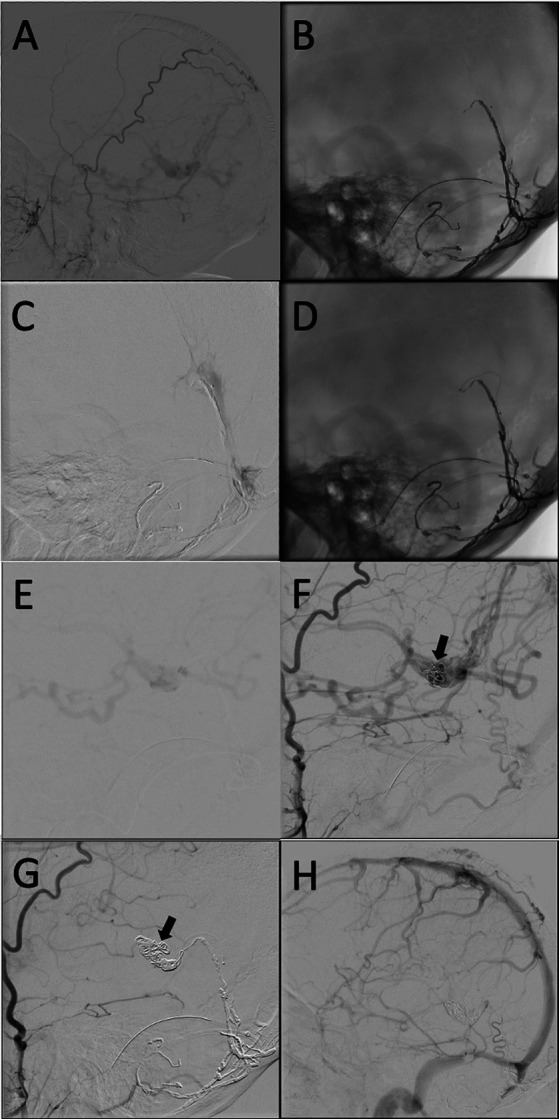
**(A)** Lateral projection of the right ECA again demonstrating an occluded straight sinus with retrograde deep drainage. **(B)** Un-subtracted lateral view showing the microcatheter in the straight sinus. **(C)** Venogram from the straight sinus showing that the vein of Galen had not been catheterized yet with delayed antegrade venous drainage through the channel created by the microcatheter. **(D)** Un-subtracted lateral view showing the microcatheter in the vein of Galen. **(E)** Venogram from the vein of Galen. **(F)** Lateral projection of the R ECA after the first coil placement (black arrow). **(G)** Lateral projection of the R ECA after the last coil (black arrow) in the late arterial phase. **(H)** Lateral projection of the R ECA after final coil placement and preservation of the internal cerebral veins with no residual shunting.

We began the transarterial embolization with superselective angiograms of the superficial temporal and occipital arteries. The most significant arterial supply to the fistula was from the left occipital artery. We were able to pass the transosseous point of the left occipital artery with the microcatheter but unable to achieve advance distally to fistulous point. Using Onyx-18 we were able to achieve partial penetration of the fistulous connection before significant reflux and cessation of antegrade Onyx penetration prevented further embolization. Follow-up superselective and external carotid angiograms demonstrated a minor reduction in arterial venous shunting (Figures 3A–D). At this point, we decided that further transarterial embolization would not be successful. She tolerated the procedure well and was discharged home the following day.

We again discussed the treatment options and ultimately recommended a transvenous approach given the diffuse arterial supply, complexity of venous drainage, largely unsuccessful transarterial embolization, morbidity of surgery, rupture risk, and delayed occlusion time with radiosurgery. Various transvenous access routes were discussed, including *via* the superior ophthalmic vein, but the navigation to the vein of Galen was thought to be challenging with a high risk for complications. We decided that access to the vein of Galen through the occluded straight sinus would carry a lower intra-operative risk if we could navigate a catheter through the occlusion.

Approaching the VoG *via* the right transverse sinus was attempted using a Navien 058 guide microcatheter over a headway duo 156 and Asahi 14. However, despite several attempts, the microcatheter could not be advanced into the VoG to the level of dAVF. Further attempts were made using a headway duo 167 advanced over an Asahi 008 microguidewire, and then with a Marathon microcatheter over the Asahi 008 microguidewire. However, each time the microcatheter could not track over the wire through the occluded straight sinus to reach the VoG. At this time, with no immediate complications, the procedure was aborted.

Two weeks later, transvenous embolization was attempted again *via* the contralateral (left) transverse sinus. A 6-French × 80 cm long shuttle sheath (Cook Medical LLC, Bloomington IN) was placed in the left Internal Jugular vein *via* a transfemoral approach. A 5F Glidecath (Terumo, Somerset NJ) was placed in the right external carotid artery through a transfemoral approach for roadmap guidance. We felt that the configuration of the left transverse sinus into the straight sinus had a more favorable angle for catheterization. A Navien 058 intermediate catheter was advanced *via* the shuttle into the left transverse sinus over an Excelsior SL-10 microcatheter and Asahi 14 microwire. The microcatheter and wire were advanced through the occluded straight sinus to the level of the vein of Galen (Figures 3B,C). Superselective venograms were obtained *via* the microcatheter to determine the level of the vein of Galen. Once through the occluded straight sinus, we were able to find an opening in the occluded connection between the vein of Galen and the straight sinus. The microcatheter and wire were then advanced distally to the level of the fistula (Figures 3D,E). Superselective venograms and right external carotid arteriograms were obtained confirming the appropriate positioning of the microcatheter just distal to the level of the fistula. Embolization was then performed in a retrograde fashion using Microplex Coils (MicroVention, Aliso Viejo CA) and Axium Coils (Medtronic, Minneapolis MN). Intermittent angiograms were obtained to evaluate for occlusion of the fistula (Figures 3F,G). Final follow-up arteriograms were obtained from the right external carotid artery (Figure 3H), left vertebral artery and left common carotid artery which demonstrated no residual arteriovenous shunting. Intravenous heparin boluses were given throughout the procedure. Given the significant reduction of overall flow through the venous system and the history of venous thrombosis, we decided to maintain a heparin infusion out of concern for cortical vein thrombosis.

The patient tolerated the procedure well and was observed in the Neuro-ICU for 24 h. We elected to convert the heparin infusion to Eliquis for 30 days. She was able to be discharged home on post-embolization day 1. Unfortunately, she presented to the emergency department on post-embolization day 2 with complaints of severe headache, nausea, and vomiting and was found to have a moderate volume intraventricular hemorrhage involving predominately the left ambient cistern, third and lateral ventricles. She was admitted to the Neuro-ICU, the Eliquis was reversed, and an external ventricular drain was placed due to progressive lethargy with an opening pressure of 28 mmHg. After a prolonged ICU course due to intracranial hypertension, the external ventricular drain was able to be removed and she was discharged to acute inpatient rehabilitation. She has since returned home and has no new neurologic deficits.

**Figure 4:**
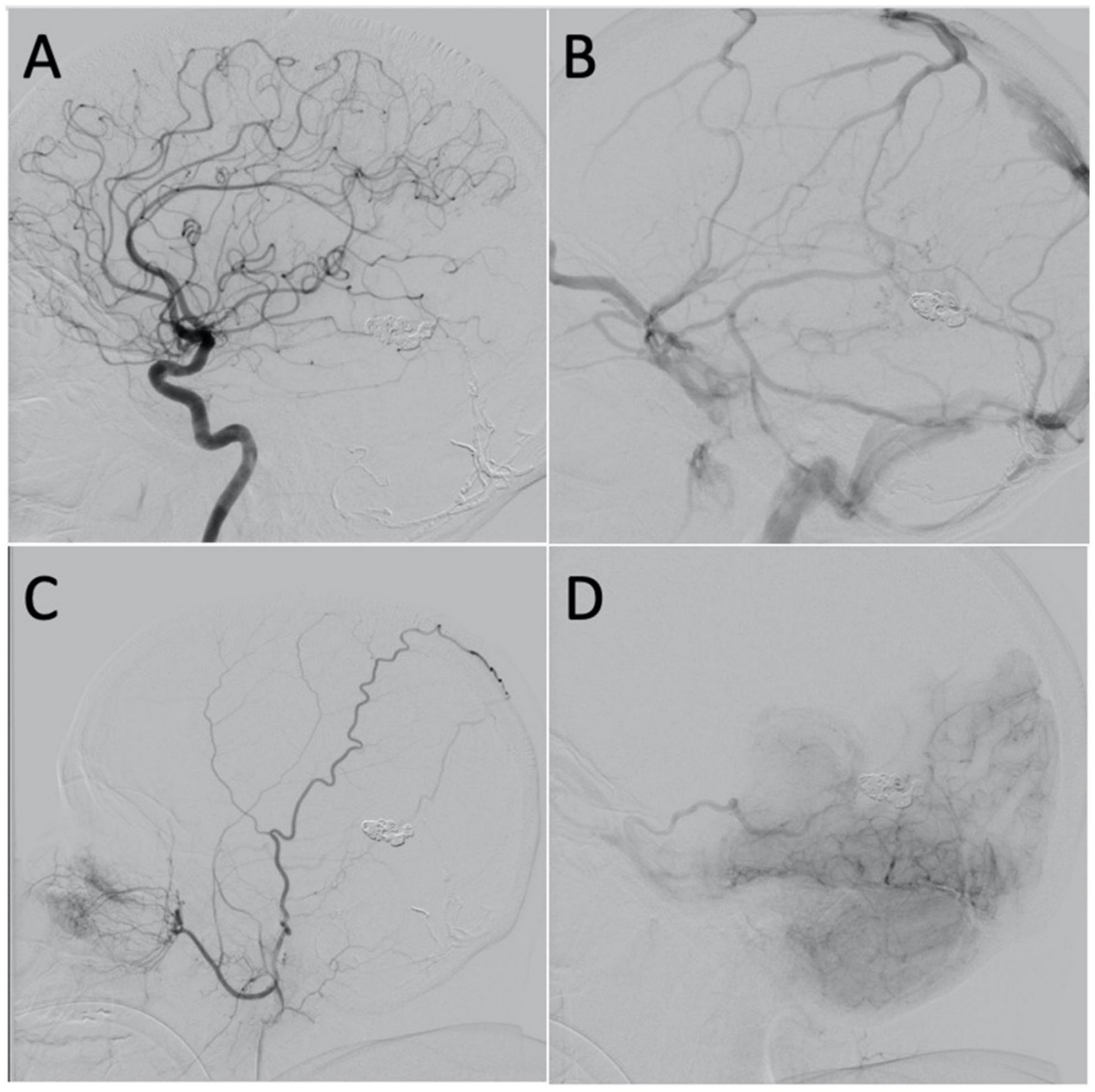
**(A)** Lateral projection of the left ICA, arterial phase, showing no shunting. **(B)** Lateral projection of the left ICA, venous phase, showing improvement in the overall venous congestion. **(C)** Lateral projection of the right ECA showing mild shunting from the falcine artery. **(D)** Lateral projection of the right vertebral artery, early venous phase, showing mild shunting from the posterior choroidal artery with venous drainage through the basal vein of Rosenthal and left superior ophthalmic vein.

A follow-up angiogram was obtained 6  months after the transvenous embolization which demonstrated significantly improved venous drainage pattern with greatly reduced venous congestion (Figures 4A–C). There was minimal residual arteriovenous shunting from the falcine artery and posterior choroidal artery with venous drainage through the basal vein of Rosenthal and left superior ophthalmic vein (Figure 4D). Clinically, she is doing remarkably well. The severe progressive headaches have resolved, and her cognitive function has improved. She is no longer experiencing “brain fog.” Given the significant angiographic and clinical improvement, we plan to repeat an angiogram in 1 year to follow the residual fistula.

## Discussion

Galenic dAVF is a rare form of falco-tentorial dAVF. Despite being the type of dAVF most associated with aggressive clinical presentation, Galenic dAVFs are infrequently described in the literature due to their overall rarity. They account for only 23% of tentorial dAVFs, with tentorial dAVFs accounting for less than 4% of all intracranial dAVFs (6, 8, 9).

Galenic dAVFs are distinct in their location at the anterior falco-tentorial junction, a location that is often obscured from surgical visualization, requiring complex operative approaches. In addition to their difficult location, their vast arterial supply and complex venous drainage, as in this patient, make them difficult to obliterate *via* endovascular approaches alone without microsurgical or radiosurgical interruption (6, 7, 10). Recent literature suggests an increase in the utilization of transarterial embolization approach in Galenic dAVFs. However, a recent report has shown that select cases can successfully be treated by the transvenous approach (8).

In our case, the absence of antegrade drainage of the VoG to the straight sinus with cortical venous reflux placed this patient at high risk for intracerebral hemorrhage. Given the hemorrhage risk and progressive symptoms, we believed that treatment was strongly indicated. Transarterial embolization was initially attempted due to promising targets at the left occipital arterial feeders, its preferred utilization in high-risk dAVFs, and an overall lower risk of intra and post-procedural hemorrhage (11). However, no significant reduction of the shunting was achieved due to the inability to reach the venous side of the fistula. In the setting of diffuse arterial supply, complexity, and lack of other safe and effective transarterial approaches, it was determined that a transvenous approach would be necessary to reach and completely occlude the fistula.

However, several veins converge into the VoG and anatomical variation can create difficulty in distinguishing them from one another, making the transvenous approach more challenging (12). The venous drainage pattern must be assessed thoroughly to find the best venous route. Venous embolization can be performed with acceptable safety if the target veins no longer contribute to normal venous drainage (8). Utilization of angiography to confirm appropriate positioning of the coils is critical in limiting the risk of unintended distal venous occlusion and residual shunting. In the case presented here, the transvenous approach permitted complete occlusion of the dAVF in a single coiling session, with significant angiographic and clinical improvement at 6 months.

## Conclusion

Galenic dAVFs are generally accepted as being one of the most difficult fistulas to approach surgically. While the transarterial embolization is typically the initial route chosen for treatment, the diffuse arterial supply of Galenic dAVFs can make this approach largely unsuccessful. Our case demonstrates the feasibility of transvenous endovascular coiling *via* an occluded straight sinus and its efficacy in treating high-grade Galenic dAVFs with retrograde venous drainage and corticovenous reflux.

### Patient perspective

“In the spring of 2021 I went to my eye Dr. to get my eyes checked. My Dr. noticed that both of my optic nerves were swollen. I was then scheduled for an MRI where it showed a vein was enlarged and was scheduled for an angiogram of the brain. It showed I had a fistula and that I had problems with back flow of those veins. I then had two veins glued on the left side of my brain. After that procedure, I was scheduled for another angiogram, but it was unsuccessful. I was then scheduled for another angiogram where they put in seven coils in my brain. I stayed overnight and was sent home. The next day, I was very sick and had a really bad headache. My husband called 911 and I was taken to UNC Hospital’s emergency room. I had bilateral bleeds. I do not know what all happened except I was put in a medically induced coma. The first day I was coherent I was talking to my husband and sister about what had happened to me. I went from ICU to a step-down unit then to rehab. I came home with the only thing wrong with me is a drop foot on the left side.”

## Data availability statement

The original contributions presented in the study are included in the article/supplementary material, further inquiries can be directed to the corresponding author.

## Ethics statement

Written informed consent was obtained from the individual(s) for the publication of any potentially identifiable images or data included in this article.

## Author contributions

CC and NQ wrote the original draft of the manuscript. CC, NQ, EY, and SS conceptualized, analyzed, reviewed, edited, and approved the published version of this manuscript. NQ, EY, and SS were treating physicians of the patient. All authors contributed to the article and approved the submitted version.

## Conflict of interest

The authors declare that this report was conducted in the absence of any commercial or financial relationships that could be construed as a potential conflict of interest.

## Publisher’s note

All claims expressed in this article are solely those of the authors and do not necessarily represent those of their affiliated organizations, or those of the publisher, the editors and the reviewers. Any product that may be evaluated in this article, or claim that may be made by its manufacturer, is not guaranteed or endorsed by the publisher.
